# 2-{5-[*N*-(2-Pyridyl)carbamo­yl]pentan­amido}pyridinium hexa­fluoro­phosphate

**DOI:** 10.1107/S1600536809026488

**Published:** 2009-07-11

**Authors:** Pei-Chi Cheng, Chia-Jun Wu, Huan-Ching Chen, Jhy-Der Chen, Ju-Chun Wang

**Affiliations:** a Department of Chemistry, Chung-Yuan Christian University, Chung-Li, Taiwan; b Department of Chemistry, Soochow University, Taipei, Taiwan

## Abstract

In the crystal structure of the title compound, C_16_H_19_N_4_O_2_^+^·PF_6_^−^, the cations and anions are situated on centres of inversion. Thus, the N—H H atom is disordered over both N atoms due to symmetry. In the crystal, mol­ecules are connected *via* N—H⋯F and N—H⋯O hydrogen bonds. The cation adopts the ⋯*AAA*⋯ *trans* conformation in the solid state.

## Related literature

For similar structures, see: Chen *et al.* (2007 ▶).
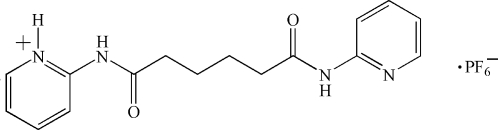


## Experimental

### 

#### Crystal data

C_16_H_19_N_4_O_2_^+^·PF_6_^−^
*M* *_r_* = 444.32
Monoclinic, 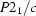

*a* = 6.2119 (18) Å
*b* = 12.9265 (11) Å
*c* = 11.439 (2) Å
β = 96.415 (10)°
*V* = 912.8 (3) Å^3^
*Z* = 2
Mo *K*α radiation
μ = 0.23 mm^−1^
*T* = 295 K
0.5 × 0.2 × 0.2 mm

#### Data collection

Bruker P4 diffractometer
Absorption correction: multi-scan (*XSCANS*; Siemens, 1995 ▶) *T*_min_ = 0.945, *T*_max_ = 0.962
2288 measured reflections
1612 independent reflections
1334 reflections with *I* > 2σ(*I*)
*R*_int_ = 0.020
3 standard reflections every 97 reflections intensity decay: none

#### Refinement

*R*[*F* ^2^ > 2σ(*F* ^2^)] = 0.036
*wR*(*F* ^2^) = 0.096
*S* = 1.07
1612 reflections
133 parameters
H-atom parameters constrained
Δρ_max_ = 0.40 e Å^−3^
Δρ_min_ = −0.30 e Å^−3^

### 

Data collection: *XSCANS* (Siemens, 1995 ▶); cell refinement: *XSCANS*; data reduction: *SHELXTL* (Sheldrick, 2008 ▶); program(s) used to solve structure: *SHELXS97* (Sheldrick, 2008 ▶); program(s) used to refine structure: *SHELXL97* (Sheldrick, 2008 ▶); molecular graphics: *SHELXTL*; software used to prepare material for publication: *SHELXTL*.

## Supplementary Material

Crystal structure: contains datablocks I, global. DOI: 10.1107/S1600536809026488/nc2150sup1.cif

Structure factors: contains datablocks I. DOI: 10.1107/S1600536809026488/nc2150Isup2.hkl

Additional supplementary materials:  crystallographic information; 3D view; checkCIF report

## Figures and Tables

**Table 1:** Hydrogen-bond geometry (Å, °)

*D*—H⋯*A*	*D*—H	H⋯*A*	*D*⋯*A*	*D*—H⋯*A*
N1—H1*A*⋯F1	0.86	1.98	2.737 (2)	145
N1—H1*A*⋯O	0.86	2.10	2.674 (2)	124
N2—H2*A*⋯F3^i^	0.86	1.95	2.774 (2)	161
N2—H2*A*⋯F1^i^	0.86	2.40	3.050 (2)	133

Symmetry code: (i) 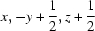.

**Fig. 1.**
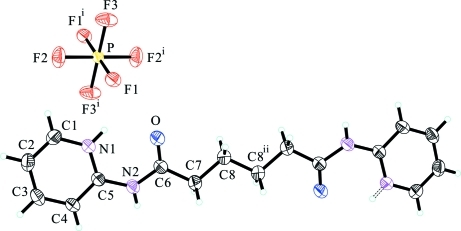
The title molecule with the labelling scheme. The bond to the disordered H atom is indicated by dashed open lines. The displacement ellipsoids are drawn at the 30% probability level.Symmetry codes: (i) -x,-y+1,-z; (ii) -x-1,-y,-z.

## supplementary crystallographic information

### Comment

The compound *N^1^*,*N^2^*-di(2-pyridyl)adipoamide has been used as bridging ligand in coordination chemistry (Chen *et al.*, 2007). In the present work the structure of the title compound (Fig. 1) has been determined to investigate the role of the cation-anion interaction on the ligand conformation. The molecules are connected *via* N—H···F and N—H···O hydrogen bonds (Tab. 1). The cation adopts the AAA *trans* conformation in the solid state. This conformation is the same as that found for the neutral *N^1^*,*N^2^*-di(2-pyridyl)adipoamide ligand which cocrystallize with water (Chen *et al.*, 2007).

### Experimental

*N^1^*,*N^2^*-Di(2-pyridyl)adipoamide (0.30 g, 1.00 mmol) and AgPF_6_ (0.25 g, 1.00 mmol) were placed in a flask containing 20 ml of CH_2_Cl_2_. The mixture was refluxed for 8 h to give a white precipitate, which was then filtered and dried under vacuum. By dissolving the solid in dichloromethane, followed by allowing the solvent to evaporate slowly under air, plate colorless crystals suitable for X-ray crystallography were obtained.

### Refinement

All the hydrogen atoms were situated into idealized positions and constrained by the riding atom approximation with *C*—H = 0.93 — 0.97 Å, N—H = 0.86 Å and *U*_iso_(H) = 1.2*U*_eq_(C, N). The occupancy of the H atom H1A was set to be 0.5 to balance the charge. Because of the disorder of the N-H H atom, the structure was also refined in space group *P*c and *P*2_1_. However, even in these cases the disorder is still present and therefore, space group *P*2_1_/*c* was selected.

### Crystal data

**Table:**

C_16_H_19_N_4_O_2_^+^·PF_6_^−^	*F*(000) = 456
*M**_r_* = 444.32	*D*_x_ = 1.617 Mg m^−^^3^
Monoclinic, *P*2_1_/*c*	Mo *K*α radiation, λ = 0.71073 Å
Hall symbol: -P 2ybc	Cell parameters from 27 reflections
*a* = 6.2119 (18) Å	θ = 5.1–12.5°
*b* = 12.9265 (11) Å	µ = 0.23 mm^−^^1^
*c* = 11.439 (2) Å	*T* = 295 K
β = 96.415 (10)°	Plate, colorless
*V* = 912.8 (3) Å^3^	0.5 × 0.2 × 0.2 mm
*Z* = 2	

### Data collection

**Table:**

Bruker P4 diffractometer	1334 reflections with *I* > 2σ(*I*)
Radiation source: fine-focus sealed tube	*R*_int_ = 0.020
graphite	θ_max_ = 25.0°, θ_min_ = 2.4°
ω scans	*h* = −1→7
Absorption correction: multi-scan (*XSCANS*; Siemens, 1995)	*k* = −15→1
*T*_min_ = 0.945, *T*_max_ = 0.962	*l* = −13→13
2288 measured reflections	3 standard reflections every 97 reflections
1612 independent reflections	intensity decay: none

### Refinement

**Table:**

Refinement on *F*^2^	Primary atom site location: structure-invariant direct methods
Least-squares matrix: full	Secondary atom site location: difference Fourier map
*R*[*F*^2^ > 2σ(*F*^2^)] = 0.036	Hydrogen site location: inferred from neighbouring sites
*wR*(*F*^2^) = 0.096	H-atom parameters constrained
*S* = 1.07	*w* = 1/[σ^2^(*F*_o_^2^) + (0.0403*P*)^2^ + 0.4314*P*] where *P* = (*F*_o_^2^ + 2*F*_c_^2^)/3
1612 reflections	(Δ/σ)_max_ < 0.001
133 parameters	Δρ_max_ = 0.40 e Å^−^^3^
0 restraints	Δρ_min_ = −0.30 e Å^−^^3^

### Special details

**Table:**

Experimental. Refinement of *F*^2^ against ALL reflections. The weighted *R*-factor *wR* and goodness of fit *S* are based on *F*^2^, conventional *R*-factors *R* are based on *F*, with *F* set to zero for negative *F*^2^. The threshold expression of *F*^2^ > σ(*F*^2^) is used only for calculating *R*-factors(gt) *etc*. and is not relevant to the choice of reflections for refinement. *R*-factors based on *F*^2^ are statistically about twice as large as those based on *F*, and *R*- factors based on ALL data will be even larger.
Geometry. All e.s.d.'s (except the e.s.d. in the dihedral angle between two l.s. planes) are estimated using the full covariance matrix. The cell e.s.d.'s are taken into account individually in the estimation of e.s.d.'s in distances, angles and torsion angles; correlations between e.s.d.'s in cell parameters are only used when they are defined by crystal symmetry. An approximate (isotropic) treatment of cell e.s.d.'s is used for estimating e.s.d.'s involving l.s. planes.
Refinement. Refinement of *F*^2^ against ALL reflections. The weighted *R*-factor *wR* and goodness of fit *S* are based on *F*^2^, conventional *R*-factors *R* are based on *F*, with *F* set to zero for negative *F*^2^. The threshold expression of *F*^2^ > σ(*F*^2^) is used only for calculating *R*-factors(gt) *etc*. and is not relevant to the choice of reflections for refinement. *R*-factors based on *F*^2^ are statistically about twice as large as those based on *F*, and *R*- factors based on ALL data will be even larger.

### Fractional atomic coordinates and isotropic or equivalent isotropic displacement parameters (Å^2^)

**Table:**

	*x*	*y*	*z*	*U*_iso_*/*U*_eq_	Occ. (<1)
P	0.0000	0.5000	0.0000	0.0275 (2)	
F1	0.0589 (2)	0.37673 (9)	−0.03443 (10)	0.0367 (3)	
F2	0.2624 (2)	0.52790 (12)	0.01505 (15)	0.0549 (4)	
F3	−0.0162 (3)	0.53230 (11)	−0.14390 (11)	0.0517 (4)	
O	−0.0438 (3)	0.16414 (14)	0.01248 (14)	0.0526 (5)	
N1	0.2814 (3)	0.26186 (14)	0.14024 (15)	0.0331 (4)	
H1A	0.1777	0.2752	0.0861	0.040*	0.50
N2	0.0760 (3)	0.12150 (15)	0.20013 (15)	0.0352 (4)	
H2A	0.0473	0.0858	0.2599	0.042*	
C1	0.4629 (4)	0.32123 (18)	0.1488 (2)	0.0387 (5)	
H1B	0.4739	0.3749	0.0957	0.046*	
C2	0.6283 (4)	0.30293 (19)	0.2341 (2)	0.0410 (5)	
H2B	0.7533	0.3430	0.2392	0.049*	
C3	0.6078 (4)	0.22297 (19)	0.3140 (2)	0.0386 (5)	
H3A	0.7187	0.2102	0.3739	0.046*	
C4	0.4248 (4)	0.16336 (18)	0.30451 (18)	0.0347 (5)	
H4A	0.4107	0.1102	0.3579	0.042*	
C5	0.2600 (3)	0.18256 (16)	0.21465 (17)	0.0300 (5)	
C6	−0.0652 (4)	0.11249 (17)	0.09912 (19)	0.0336 (5)	
C7	−0.2390 (4)	0.03259 (18)	0.10327 (19)	0.0365 (5)	
H7A	−0.3056	0.0409	0.1755	0.044*	
H7B	−0.1743	−0.0357	0.1041	0.044*	
C8	−0.4124 (4)	0.04088 (18)	−0.0011 (2)	0.0375 (5)	
H8A	−0.4784	0.1089	−0.0012	0.045*	
H8B	−0.3451	0.0339	−0.0733	0.045*	

### Atomic displacement parameters (Å^2^)

**Table:**

	*U*^11^	*U*^22^	*U*^33^	*U*^12^	*U*^13^	*U*^23^
P	0.0269 (4)	0.0280 (4)	0.0265 (4)	−0.0007 (3)	−0.0013 (3)	−0.0024 (3)
F1	0.0443 (7)	0.0275 (6)	0.0364 (6)	0.0055 (6)	−0.0032 (5)	−0.0043 (5)
F2	0.0268 (7)	0.0474 (8)	0.0887 (12)	−0.0045 (6)	−0.0011 (7)	−0.0043 (8)
F3	0.0854 (11)	0.0420 (8)	0.0276 (7)	0.0119 (7)	0.0052 (7)	0.0018 (6)
O	0.0577 (12)	0.0553 (11)	0.0398 (9)	−0.0212 (9)	−0.0163 (8)	0.0162 (8)
N1	0.0326 (10)	0.0340 (10)	0.0319 (9)	−0.0024 (8)	−0.0009 (8)	0.0032 (8)
N2	0.0315 (10)	0.0448 (11)	0.0282 (9)	−0.0073 (9)	−0.0020 (7)	0.0074 (8)
C1	0.0428 (13)	0.0332 (12)	0.0395 (12)	−0.0057 (10)	0.0023 (10)	0.0022 (10)
C2	0.0328 (12)	0.0410 (13)	0.0482 (13)	−0.0077 (10)	−0.0002 (10)	−0.0053 (11)
C3	0.0307 (11)	0.0445 (14)	0.0381 (12)	0.0037 (10)	−0.0077 (9)	−0.0038 (10)
C4	0.0333 (12)	0.0385 (12)	0.0305 (10)	0.0014 (10)	−0.0040 (9)	0.0043 (9)
C5	0.0277 (11)	0.0336 (11)	0.0281 (10)	0.0009 (9)	0.0007 (8)	0.0005 (9)
C6	0.0325 (11)	0.0342 (11)	0.0329 (11)	0.0008 (9)	−0.0022 (9)	0.0012 (9)
C7	0.0367 (12)	0.0382 (12)	0.0330 (11)	−0.0049 (10)	−0.0023 (9)	0.0004 (10)
C8	0.0353 (11)	0.0362 (12)	0.0394 (12)	−0.0046 (10)	−0.0024 (10)	0.0009 (10)

### Geometric parameters (Å, °)

**Table:**

P—F2^i^	1.6596 (14)	C1—H1B	0.9300
P—F2	1.6596 (14)	C2—C3	1.395 (3)
P—F3	1.6902 (13)	C2—H2B	0.9300
P—F3^i^	1.6902 (13)	C3—C4	1.368 (3)
P—F1	1.6912 (12)	C3—H3A	0.9300
P—F1^i^	1.6913 (12)	C4—C5	1.389 (3)
O—C6	1.214 (3)	C4—H4A	0.9300
N1—C5	1.348 (3)	C6—C7	1.499 (3)
N1—C1	1.358 (3)	C7—C8	1.520 (3)
N1—H1A	0.8600	C7—H7A	0.9700
N2—C6	1.375 (3)	C7—H7B	0.9700
N2—C5	1.383 (3)	C8—C8^ii^	1.519 (4)
N2—H2A	0.8600	C8—H8A	0.9700
C1—C2	1.356 (3)	C8—H8B	0.9700
			
F2^i^—P—F2	180.0	C3—C2—H2B	120.5
F2^i^—P—F3	90.09 (8)	C4—C3—C2	120.1 (2)
F2—P—F3	89.91 (8)	C4—C3—H3A	120.0
F2^i^—P—F3^i^	89.91 (8)	C2—C3—H3A	120.0
F2—P—F3^i^	90.09 (8)	C3—C4—C5	119.7 (2)
F3—P—F3^i^	180.00 (9)	C3—C4—H4A	120.2
F2^i^—P—F1	90.39 (7)	C5—C4—H4A	120.2
F2—P—F1	89.61 (7)	N1—C5—N2	119.76 (18)
F3—P—F1	89.82 (6)	N1—C5—C4	119.09 (19)
F3^i^—P—F1	90.18 (6)	N2—C5—C4	121.14 (19)
F2^i^—P—F1^i^	89.61 (7)	O—C6—N2	121.4 (2)
F2—P—F1^i^	90.39 (7)	O—C6—C7	123.3 (2)
F3—P—F1^i^	90.18 (6)	N2—C6—C7	115.21 (18)
F3^i^—P—F1^i^	89.82 (6)	C6—C7—C8	112.12 (18)
F1—P—F1^i^	180.00 (8)	C6—C7—H7A	109.2
C5—N1—C1	121.58 (19)	C8—C7—H7A	109.2
C5—N1—H1A	119.2	C6—C7—H7B	109.2
C1—N1—H1A	119.2	C8—C7—H7B	109.2
C6—N2—C5	126.15 (18)	H7A—C7—H7B	107.9
C6—N2—H2A	116.9	C8^ii^—C8—C7	112.6 (2)
C5—N2—H2A	116.9	C8^ii^—C8—H8A	109.1
C2—C1—N1	120.6 (2)	C7—C8—H8A	109.1
C2—C1—H1B	119.7	C8^ii^—C8—H8B	109.1
N1—C1—H1B	119.7	C7—C8—H8B	109.1
C1—C2—C3	118.9 (2)	H8A—C8—H8B	107.8
C1—C2—H2B	120.5		

Symmetry codes: (i) −*x*, −*y*+1, −*z*; (ii) −*x*−1, −*y*, −*z*.

### Hydrogen-bond geometry (Å, °)

**Table:**

*D*—H···*A*	*D*—H	H···*A*	*D*···*A*	*D*—H···*A*
N1—H1A···F1	0.86	1.98	2.737 (2)	145
N1—H1A···O	0.86	2.10	2.674 (2)	124
N2—H2A···F3^iii^	0.86	1.95	2.774 (2)	161
N2—H2A···F1^iii^	0.86	2.40	3.050 (2)	133

Symmetry codes: (iii) *x*, −*y*+1/2, *z*+1/2.
